# 3MD for Chronic Conditions, a Model for Motivational mHealth Design: Embedded Case Study

**DOI:** 10.2196/11631

**Published:** 2018-08-24

**Authors:** Guido Giunti

**Affiliations:** ^1^ Salumedia Tecnologias Seville Spain; ^2^ University of Oulu Oulu Finland

**Keywords:** chronic conditions, consumer health informatics, gamification, health behavioral change, medical informatics, mHealth, user-centered design, information systems

## Abstract

**Background:**

Chronic conditions are the leading cause of death in the world. Major improvements in acute care and diagnostics have created a tendency toward the chronification of formerly terminal conditions, requiring people with these conditions to learn how to self-manage. Mobile technologies hold promise as self-management tools due to their ubiquity and cost-effectiveness. The delivery of health-related services through mobile technologies (mobile health, mHealth) has grown exponentially in recent years. However, only a fraction of these solutions take into consideration the views of relevant stakeholders such as health care professionals or even patients. The use of behavioral change models (BCMs) has proven important in developing successful health solutions, yet engaging patients remains a challenge. There is a trend in mHealth solutions called gamification that attempts to use game elements to drive user behavior and increase engagement. As it stands, designers of mHealth solutions for behavioral change in chronic conditions have no clear way of deciding what factors are relevant to consider.

**Objective:**

The goal of this work is to discover factors for the design of mHealth solutions for chronic patients using negotiations between medical knowledge, BCMs, and gamification.

**Methods:**

This study uses an embedded case study research methodology consisting of 4 embedded units: 1) cross-sectional studies of mHealth applications; 2) statistical analysis of gamification presence; 3) focus groups and interviews to relevant stakeholders; and 4) research through design of an mHealth solution. The data obtained was thematically analyzed to create a conceptual model for the design of mHealth solutions.

**Results:**

The Model for Motivational Mobile-health Design (3MD) for chronic conditions guides the design of condition-oriented gamified behavioral change mHealth solutions. The main components are (1) condition specific, which describe factors that need to be adjusted and adapted for each particular chronic condition; (2) motivation related, which are factors that address how to influence behaviors in an engaging manner; and (3) technology based, which are factors that are directly connected to the technical capabilities of mobile technologies. The 3MD also provides a series of high-level illustrative design questions for designers to use and consider during the design process.

**Conclusions:**

This work addresses a recognized gap in research and practice, and proposes a unique model that could be of use in the generation of new solutions to help chronic patients.

## Introduction

### Background

Chronic conditions are by far the leading cause of mortality in the world, representing more than 60% of all deaths [1] and taking more and more precedence over “traditional” acute illnesses. This is in part due to the increased average life expectancy [2] and major improvements in acute care and diagnostics that have created a tendency toward the chronification of formerly terminal conditions [3,4]. In these conditions, care is shifting to outpatient settings requiring people to learn how to manage on their own [5]. Chronic condition self-management refers to the ability of an individual, in conjunction with family, community, and health care professionals, to manage symptoms, treatments, lifestyle changes, and psychosocial and cultural consequences of health conditions [6]. Studies show that behavior patterns are among the main determinants of health, with actual health care services following far behind the individual’s social circumstances [7]. The fact that behavioral change is still a great barrier for patients is a recognized problem [8].

The field of consumer health informatics researches the role of information technology (IT) for health care consumers. Consumer health informatics is defined by Gunther Eysenbach as a field that “analyzes consumers’ needs for information, studies and implements methods of making information accessible to consumers, and models and integrates consumers’ preferences into medical information systems” [9]. Consumer health informatics can play a vital role for patient engagement and patient empowerment as it allows patients to take charge of their own health and their interactions with health professionals [10,11]. In this sense, mobile technologies hold promise because of their ubiquity, cost-effectiveness, less invasive nature, and their ability to provide immediate feedback and track activities [12-14]. By 2017, the global use of mobile phones had reached over 3.2 billion devices [15] allowing a variety of health interventions. The delivery of health-related services through mobile technologies and other wearable devices is called mHealth (mobile health) [16].

The use of mHealth mobile software apps has grown exponentially in recent years [17], with more than 100,000 apps available for download on online stores [18]. The world is currently seeing a surge of digital health start-ups [19] whose mHealth solutions usually fall into the general wellness, exercise, and diet category [20], neglecting condition-specific services. Only a fraction of these apps and services take into consideration the perspectives of relevant stakeholders, such as health care professionals and sometimes even patients themselves. To this purpose, a design philosophy called user-centered design (UCD) could prove useful because it places the needs and characteristics of intended users first and foremost [21-23]. In this manner, the goal of UCD is to create solutions specific to the user and the intended tasks [22,23]. Following UCD principles can generate systems that are easy to learn, have higher user acceptance and satisfaction, and lower user errors [22-24]. In addition, following good design principles early on not only can save time and money [25], but also decreases design changes late in the development process [24,26]. The use of UCD has been gaining traction in the design of health IT solutions, but it still is in its infancy.

A recent meta-analysis of behavioral change interventions showed that theory-based approaches have greater impact [27]. However, finding ways that engage patients to continue with an intervention is still a difficult task [28]. Additionally, in the past few years, more mHealth solutions have begun to use game elements to drive user behavior [29] in a practice known as *gamification* [30]. Game elements are incorporated into the greater context of the mobile app to bolster usability and compel continued and prolonged use [31,32]. However, gamification is not thoroughly understood yet. Despite the existence of some health gamification frameworks, a systematic review [33] found that as far as gamification design frameworks are concerned, the health sector is the least developed.

As it stands, designers of mHealth solutions for chronic conditions, who intend to create behavioral change interventions and integrate motivational elements, have no clear way of deciding what the relevant factors to consider are. This presents a relevant gap in knowledge that is yet to be answered appropriately in this field of study. The goal of this work is to address the lack of a model that allows the integration of motivational elements in the design mHealth solutions for chronic conditions.

### Related Works

This section presents the theoretical background and scientific works related to this paper. Relevant medical concepts, behavioral change theories, and gamification considerations are described.

#### Chronic Conditions

Chronic conditions have a course that varies over time that is specific to the particular illness and can be very intrusive to everyday life. However, some common challenges across managing chronic conditions exist, such as recognizing symptoms and taking appropriate actions, handling complex treatment regimens, developing coping strategies, and dealing with frequent interactions with the health care system over time [34].

The context of this study (see Setting) provided the opportunity to work on two very different conditions: breast cancer and multiple sclerosis (MS).

Breast cancer is the most common cancer in women both in the developed and less developed world [1]. Thanks to advancements in treatments, breast cancer survivorship is on a steady rise and this cancer is no longer thought of as an acute illness but rather a chronic condition [3,4]. It is common to find mHealth solutions for breast cancer in the scientific literature such as tracking sleep patterns [35], symptoms and treatment side effect management [35-37], breast health and well-being assessments [38,39], and even comprehensive lifestyle programs with nutrition and physical activity elements [40].

MS is one of the world’s most common neurologic disorders [41]. The most common symptoms are overwhelming fatigue, visual disturbances, altered sensation, cognitive problems, and difficulties with mobility [42]. There have been recommendations that suggest the incorporation of standard MS management tools into mHealth solutions [43], and the scientific literature shows that some health apps do exist for fatigue assessment and fatigue management [44], emotional support [45], or self-management [46].

#### Behavioral Change

There are several theories and behavioral change models (BCMs) that are used in health behavior science with the main goal of making the healthy choice the easy choice.

The use of computerized health behavior interventions has expanded rapidly in the last decade and existing BCMs have been used to guide mHealth interventions: There is a growing body of evidence suggesting that mHealth can support health behavioral change in areas such as smoking cessation, physical activity, and other health care problems [47-51].

The use of instant feedback and positive reinforcement from learning theories are in common use in mHealth apps [29,47]. The Health Belief Model has been used in mHealth interventions for self-management and health promotion [52-54], the Transtheoretical Model has been used in mobile solutions for smoking cessation and other addictive behaviors [55-58], and physical activity and fitness interventions use the theory of planned behavior [29,50,59] as well as self-regulation theories [29,60-63]. The basis for social cognitive theories can be found in many interventions using health apps for disease management [64-66] and goal setting is very often used in mHealth apps [60,67]. It has been noted that each BCM carries its limitations and problems [68-70]. A multitheory approach is usually recommended in behavioral change intervention design [71] and this should be considered when designing mHealth solutions.

Mobile devices have the capacity to interact with the individual with much greater frequency and in the context of the behavior [72]. mHealth interventions allow for tailoring not only during the beginning of an intervention process, but also during the course of intervention [73]. As such, these mobile technologies are “always on” and are carried on the person throughout the day, offering more chances for interaction and intervention [17]. Therefore, mHealth interventions for behavioral change would benefit from contemplating the dynamic nature that mobile capabilities have to offer: rapid intervention adaptation based on the individual’s current and past behavior and situational context [17]. A behavior change support system (BCSS) is a sociotechnical information system with psychological and behavioral outcomes designed to form, alter, or reinforce attitudes, behaviors, or an act of complying without using coercion or deception [48]. The creation of BCSS involves a variety of disciplines from human sciences to information systems.

There are BCSS design models such as the Persuasive Systems Design (PSD) [74], which concerns the design of persuasive technologies in general. In this model, the need for recognizing the intent of persuasion, understanding the persuasion event, and defining and/or recognizing the strategies in use are key. Another BCSS design model is the IDEAS (Integrate, Design, Assess, and Share) framework [75]. In this model, behavioral change theory and design thinking are integrated to guide the development of digital health interventions. The Chronic Disease mHealth App Intervention Design Framework [76] is specific to mHealth and it focuses on chronic conditions, addressing issues present in the other frameworks. The issue of enjoying doing the behavior, however, is not addressed in these models.

#### Gamification

It is not surprising that efforts to translate the feeling of engagement and enjoyment that games have to other areas of our life have been attempted. Gamification is generally understood as the use of game elements in nongame contexts [30] and its use can be seen as one form of persuasive or motivational design [77].

In this work, the terms *gamification design* and *gameful design* are used interchangeably, since they frame the same extension of phenomena through different intentional properties [78].

##### Gamification Elements

Game elements are varied, but usually the literature on game design considers the following to be the basic set [78-80]:

1. Points and leveling systems, which provide feedback and inform the user of their level of familiarity of the system.
2. Leaderboards that are used to dynamically rank individual user progress and achievements as compared to their peers.
3. Badges, achievements, and trophies, which act as rewards for the accomplishment of specific tasks.
4. Challenges and quests that constitute objectives and create a narrative within the system.
5. Social features are used to support and reinforce interaction between users.

Each of these elements by themselves are not seen as “gameful” [78], but combined and arranged in certain ways, they can tap into something greater and unlock a unique experience. In the context of mobile apps, these elements are integrated as specific features for purposes of bolstering usability and compelling continued use [31,32].

##### Users and Player Types

As with BCMs, the literature suggests that the different user or player types will have different needs and it could be useful to keep them in mind during the design process. Asking gamers why they play videogames shows that there is no single and unified answer [81].

There have been many attempts to create “player types” for design and analysis purposes. Game designer Richard Bartle observed the way users of an online game behaved and wrote down his observations creating what is now known as Bartle’s taxonomy [82]. However, Bartle’s taxonomy was never intended to be a general typology, only a description of his observations in one particular context [83]. Others have tried to address this problem, such as Yee [84] with his empirical model of player motivations, or Marczewski [85] who developed the Gamification User Types Hexad framework using self-determination theory as the theoretical background and research on human motivation, player types, and practical design experience. According to Marczewski, user types are segmented and supported in the following ways:

1. Philanthropists are individuals motivated by altruistic purposes, willing to give without expecting a reward within the system.
2. Socializers want to interact with others and create social connections. The system is important to them but as a means to connect.
3. Free Spirits desire the freedom to express themselves and act without external control. They like to create and explore within a system.
4. Achievers seek to progress their status by completing tasks or prove themselves by tackling difficult challenges. The system is a challenge to be overcome.
5. Players are motivated by extrinsic rewards. The specific type of reward is not important, only that the system is providing it.
6. Disruptors enjoy testing the limits of the system, looking to push past them. Sometimes they can be negative agents, sometimes their work improves the system.

##### Gameful Design Models

Gameful design is about intentionally designing for gamefulness in the development of nongame environments using game design thinking [78]. Simply inserting the different game elements into any nongame context is not sufficient—the tasks themselves have to be designed in a manner similar to game design [86]. In this sense, game design should be approached as a lens to improve the overall experience of the task.

There are models for game design such as the Mechanics, Dynamics and Aesthetics framework [87] that aim to help game designers. Designers have used this kind of game design model before [33] to gamify activities, but it is clear that the process of gameful design is somewhat different from game design. Games are mostly directed toward pure entertainment, whereas gamification attempts to enhance engagement and user experience in different contexts [88]. The design approach of a gameful system is different than that of a conventional game.

The gamification framework of the Werbach and Hunter [89] gamification framework, commonly known as 6D, is one of the most popular and referenced gamification design frameworks, created with the purpose of designing a service or product with business goals. Another commonly used framework is called Octalysis [90]. In this framework, the design process is viewed from a “human-focused” lens as opposed to “function-focused” points of view. The authors propose that design processes concentrate normally on optimizing efficiency, getting the job done, rather than on human motivation.

Even if these gamification models exist, it is important to keep in mind that one cannot expect that they perfectly translate to health scenarios. In generic gamification models, the goal is usually to increase a certain task efficiency or improve user retention [33]. Although these may look appropriate on a surface level, there are hidden dangers inherent to health care. Generic gamification models often do not contemplate potential negative consequences. Ethics should guide the design of health technologies and recognized principles of bioethics play an important role in this process [91]. Because of these issues, specific conceptual frameworks for gamification in health are being developed.

The Wheel of Sukr is a health-specific gamification framework for assisting diabetic patients to self-manage and reinforce positive behaviors [92]. The Wheel of Sukr framework uses reward systems to motivate users toward healthy behaviors. Its theoretical basis lies in reaching the state of flow and motivation as understood by self-determination theory. Another health-oriented gamification framework is PACT (People, Aesthetics, Context, and Technology) [93], a participatory design framework for the gamification of rehabilitation systems that looks to involve all the relevant stakeholders from the beginning of a rehabilitation design process. This framework, however, does not use any behavioral change theory as foundation.

Despite the existence of some health gamification frameworks, a systematic review [33] found that as far as gamification design frameworks are concerned, the health sector is the least developed.

## Methods

### Study Design

Consumer health informatics is a complex phenomenon and the study of such phenomena can often improve with the use of methodological triangulation to generate more thorough results [94,95]. The combination of different research methods tends to decrease the weaknesses of an individual method and strengthen the outcome of the study. This work uses an embedded case study methodology to research the design of mHealth solutions.

An embedded case study is a case study containing more than one subunit of analysis [96]. The embedded case study methodology provides the means of integrating quantitative and qualitative methods into a single research study [97]. Embedded case studies explore the phenomena in terms of subunits, each focusing on different features. The data obtained from the cases are interpreted in a transformational process that relies on different methods to arrive at a perception, judgment, or evaluation [97]. In this way, a synthesis is created, resulting in new knowledge.

### Setting

This work is the result of an industrial PhD experience that took place in Salumedia Tecnologias, a digital health company in Spain, over the span of 3 years as part of a Marie Skłodowska-Curie research fellowship (see Acknowledgments).

### Study Case

To explore design factors for mHealth solutions for chronic patients, different disease courses, management, and symptomatology had to be taken into consideration. Two very different chronic conditions were selected because they represent different models of chronic conditions and provide a rich area of analysis that is useful to prevent the results from being overly specific to one condition paradigm. MS represents a chronic condition that manifests itself in the life of a patient as they become young adults [41], whereas breast cancer represents an acute condition that becomes chronic thanks to improved treatments and care [3,4].

**Figure 1:**
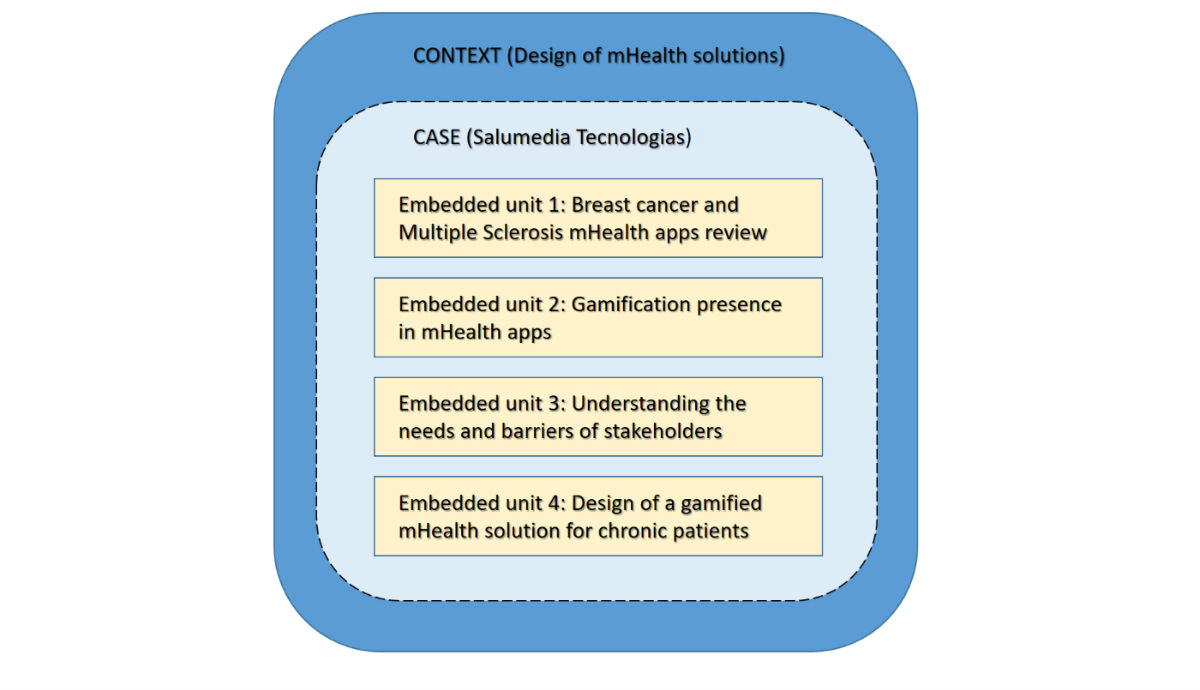
Embedded case study used in this research. The dashed lines represent the blurred boundary between a case and its context.

The way embedded case study methodology was used in this research was through a single embedded case with four embedded units as illustrated in Figure 1. The dashed lines represent the blurred boundary between a case and its context.

The design of mHealth solutions within Salumedia was used as an instrumental case study. Instrumental case studies differ in that the case itself is secondary to gaining understanding on a particular phenomenon [98]. What each embedded unit was and how it was explored is detailed in the following sections.

### Data Collection

The embedded units used different quantitative and qualitative methods which are described subsequently.

#### Embedded Unit 1: Breast Cancer and Multiple Sclerosis mHealth Apps Review

To understand the current landscape for mHealth solutions for the selected chronic conditions, two cross-sectional studies [99] were undertaken: one for breast cancer [100] and one for MS [101].

Selection criteria that would allow identification and classification of all relevant apps was designed and each app was systematically explored. Almost 600 breast cancer apps and 25 MS apps were categorized by their intended purpose, the reliability of their contents, their intended audiences, and the institutions behind each app.

There was a clear difference between MS and breast cancer apps not only in features, but also in other aspects such as their intended audiences. These studies provided insight regarding the state of the practice of mHealth solutions for chronic conditions and the features and characteristics that available health apps offer.

#### Embedded Unit 2: Gamification Presence in mHealth Apps

Gamification was present in the apps from the previous embedded unit, so the study of the phenomena continued. The larger number of breast cancer apps allowed for richer exploration.

Based on the scientific literature on gamification and game elements, and with the help of a panel of experts, we were able to generate a catalog of gamification concepts and constructs. In another study [102], we empirically studied the presence of gamification in breast cancer apps and developed a predictive model to automatically detect the presence of gamification in large samples of breast cancer health apps using only the title and description text of the app. The steps involved in the construction of this gamification screening tool nurtured the understanding of gamification techniques and how they can be applied in the design of health apps.

#### Embedded Unit 3: Understanding the Needs and Barriers of Stakeholders in Chronic Conditions

To see how UCD could be applied to the design of behavioral change mHealth solutions, we conducted a mixed methods design study with qualitative and quantitative components to explore the views of chronic patients and the health care professionals who work with them [103].

The qualitative part consisted of focus groups and interviews of persons with MS and health care professionals; the quantitative part consisted of structured surveys and standardized tools such as a satisfaction with life scale [104] and electronic health (eHealth) literacy scales [105]. Participants in this study were coded as “PWMS” for persons with MS and “HP” for health care professionals ranging from 1 to 12 (eg, PWMS11 or HP02).

The work in this study was used to inform on the design factors specific to living with and managing chronic conditions, as well as the care process involved.

#### Embedded Unit 4: Design of a Gamified mHealth Solution for Chronic Patients

Research through design is a methodology that employs methods and processes from design practice as a legitimate method of evidence [106]. In this method, design activities play a formative role in the generation of knowledge.

Building on the insight and knowledge gained from the previous embedded units and the available scientific literature, we used a research through research to design a mHealth solution called More Stamina [107]. More Stamina is a gamified fatigue management app for persons with MS. Because More Stamina attempts to create a health behavioral change, relevant models and theories were explored and understood for their use.

The design process for More Stamina required deep exploration of both BCMs and the use of gameful design. The interplay between these two areas of knowledge and the users’ needs were key during requirement negotiations that shaped the final design. The work involved in this research was valuable to the understanding of factors for mHealth design for chronic conditions.

### Data Analysis

A broad range of analysis methods were used on the collected data from both the quantitative and qualitative approaches.

#### Embedded Unit 1: Breast Cancer and Multiple Sclerosis mHealth Apps Review

The work in embedded unit 1 used a descriptive quantitative study approach to show the composition of the mHealth solutions ecosystem and what kind of tools and features are available for persons with the selected chronic conditions. For each individual study, two reviewers independently reviewed app information using structured forms and going over the app store descriptions to classify and categorize each app. Fleiss-Cohen’s coefficient was used to assess interrater reliability according to Landis and Koch’s standards [108].

#### Embedded Unit 2: Gamification Presence in mHealth Apps

In embedded unit 2, the process of creating the gamification detection algorithm used multivariate logistic regression where significant and relevant variables were incorporated into the algorithm. The reliability of the algorithm was evaluated using receiver operating characteristic (ROC) curves for the predicted values of gamification presence. Several iterations of the logistic model were compared to each other using Akaike’s information criterion (AIC) [109] and the one with the largest area under the ROC curve and the lowest AIC was selected.

#### Embedded Unit 3: Understanding the Needs and Barriers of Stakeholders in Chronic Conditions

Embedded unit 3 used two methods simultaneously: a qualitative exploration of the different stakeholders in a chronic condition and standardized structured questionnaires to complement each other. The focus groups and interviews in the qualitative part were audio-recorded, transcribed verbatim, and coded using the qualitative data analysis management program NVivo 11 (QSR International, Melbourne, Australia). The transcripts were independently analyzed first and then jointly during meetings to consolidate concepts. Recurring themes and subthemes were identified and coded during a deductive phase; thematic analysis was performed during an inductive phase [110]. The results of the quantitative standardized structured questionnaires were analyzed according to their own evaluation matrices.

#### Embedded Unit 4: Design of a Gamified mHealth Solution for Chronic Patients

The embedded unit 4 used design practice as a way of generating knowledge in an iterative and reflective manner through the practice of hands-on design work. The findings from embedded unit 3 were used as user requirements; BCMs and gamification concepts were considered during requirement negotiations. We used Nielsen’s heuristics [111] as design guidelines and evaluation methods for the usability of the resulting prototype. The evaluator team independently examined each heuristic for all prototype screens. Notes were taken on major and minor issues discovered to be later contrasted among them. After each heuristic evaluation, the prototype was modified and assessed again. This process was iterated until all usability issues were deemed to be addressed.

#### Overall Analysis

The data obtained from the different embedded units and relevant related research were then gathered for analysis. The objective of this analysis was to generate an abstraction of concepts that could be extrapolated and extracted into a series of high-level illustrative design questions. The collection of design questions was then subject to a thematic content analysis [110] in which recurring themes and subthemes were sought. This followed an inductive approach in which the themes identified were data driven. The exploration and definition of themes and subthemes focused on aspects that would be relevant in finding out how to design mHealth solutions for persons with chronic conditions, which would be valuable and meaningful for all stakeholders in the health care context and could fulfill the needs of the stakeholders. Aspects that are obvious for any information and communications technology-based solution were not incorporated; for example, the fact that the solution should be error free, that it should follow relevant laws and regulations, that cost should be within the designer’s limitations, and so on.

To help ensure the integrity of the content analyses, the guidelines set by Shenton [112] were followed, which include collecting and analyzing data in an iterative process to identify themes and generating an audit trail among others. The use of methodological triangulation allowed complementary findings to converge creating greater understanding from different parts of the different concepts.

The iterative process of grouping and subgrouping illustrative design questions led to a series of abstract constructs that were used to create a model that can be useful to guide the design process of condition-oriented gamified behavioral change mHealth solutions.

### Ethical Considerations

The ethical approval for studies involving participants was obtained from the Swiss Ethics Committee on Research Involving Humans (ID #2016-00529). The participants were informed about the nature of the research project; the reasons for their subjectability; risks, benefits, and alternatives associated with the research; and their rights as research subjects before agreeing to participate. Steps were taken to ensure that data gathered from participants were kept under strict security, anonymity, and privacy.

## Results

### A Model for Motivational mHealth Design: 3MD for Chronic Conditions

#### Overview

The embedded units were used to extract valuable insight for the study case. Data from the different embedded units and scientific literature was integrated and is presented in the subsequent sections to provide traceability and facilitate the thematic trail. As a result of the thematic analysis, design factors emerged from the data and are grouped in the form of the components for the conceptual model called “Model for Motivational Mobile-health Design (3MD) for Chronic Conditions.” A conceptual model is a high-level description of how a system is organized and operates [113]. According to Storrs [114], models are “frameworks for understanding” a subject; they are representations that are used to help people know, understand, or simulate a subject the model represents.

**Figure 2:**
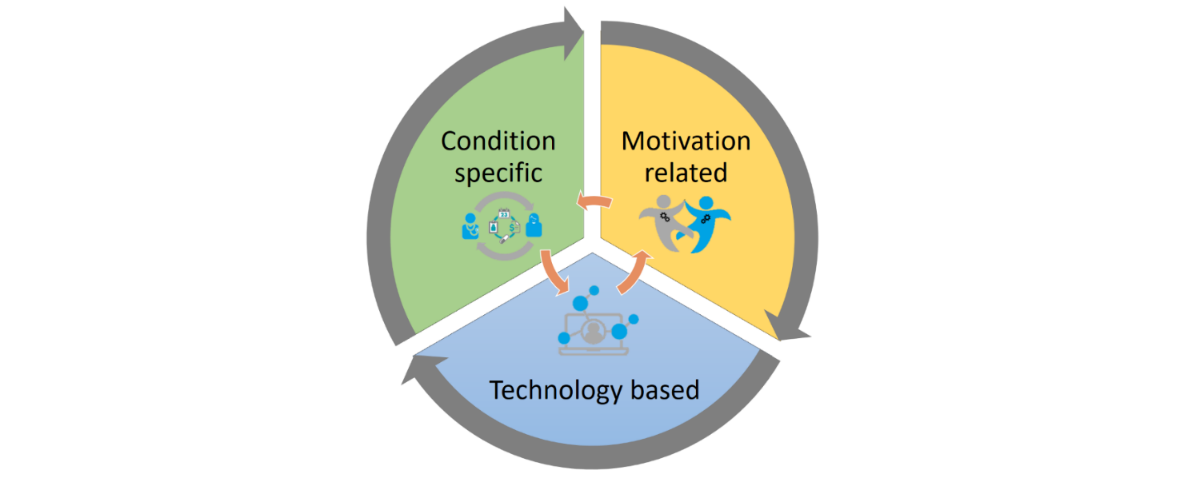
Interaction of the 3MD (Model for Motivational Mobile-health Design) components.

The main components of the 3MD for Chronic Conditions are condition specific, motivation related, and technology based (see Figure 2). A general overview of the model is presented in Textbox 1.

The 3MD is aimed at designers of mHealth solutions and because of this and the fact that the ecosystem largely consists of start-ups and individual entrepreneurs, the overall language and approach was chosen. The model proposes illustrative design questions expressed in layman’s terms, minimizing academic terminology. These questions are not definitive ones; rather, they work as a means to illustrate how to approach each component to guide the design process. Designers are encouraged to explore and expand them, creating more subsets that fit their purposes. A description of each component and their respective factors can be found in the following subsections along with their respective series of illustrative design questions.

### Condition Specific

Although chronic conditions share similar overall needs, each condition has inherent differences and idiosyncrasies. These differences require special fine tuning during design. In embedded unit 3 and embedded unit 4, the relevance of centering the design of mHealth solutions around identified patient needs and characteristics was highlighted.

The condition-specific component describes factors that need to be adjusted and adapted for the chronic condition in question. Further thematic analysis grouped these factors into subgroups: common condition problems, patient self-narrative, and care process.

#### Common Condition Problems

Persons with chronic conditions are affected by a myriad of problems that alter the way they live their lives. Some conditions require patients to spend a significant amount of time dealing with their symptoms and disease management, but these are not the only issues that ail them. The work on embedded unit 3 showed how persons with chronic conditions can be concerned or even afraid of issues that the health care team may disregard. Such was the case where one health care professional claimed:

If you ask them “how do you feel,” they will always say “I don’t feel good.” Interestingly, this feeling doesn’t change, they may train over three, four, or five weeks and they will feel the same. However, if you look at the parameters that you normally assess, you will see that they have improved. VO2, oxygen uptake, or maximum heart rate will have gone up. They objectively improve, but subjectively still feel bad. 2, oxygen uptake, or maximum heart rate will have gone up. They objectively improve, but subjectively still feel bad.

HP11

In this example, one can see that the subjective experiences of persons with MS were placed in an inferior condition than the “objective” physiological parameters.

**Figure 3:**
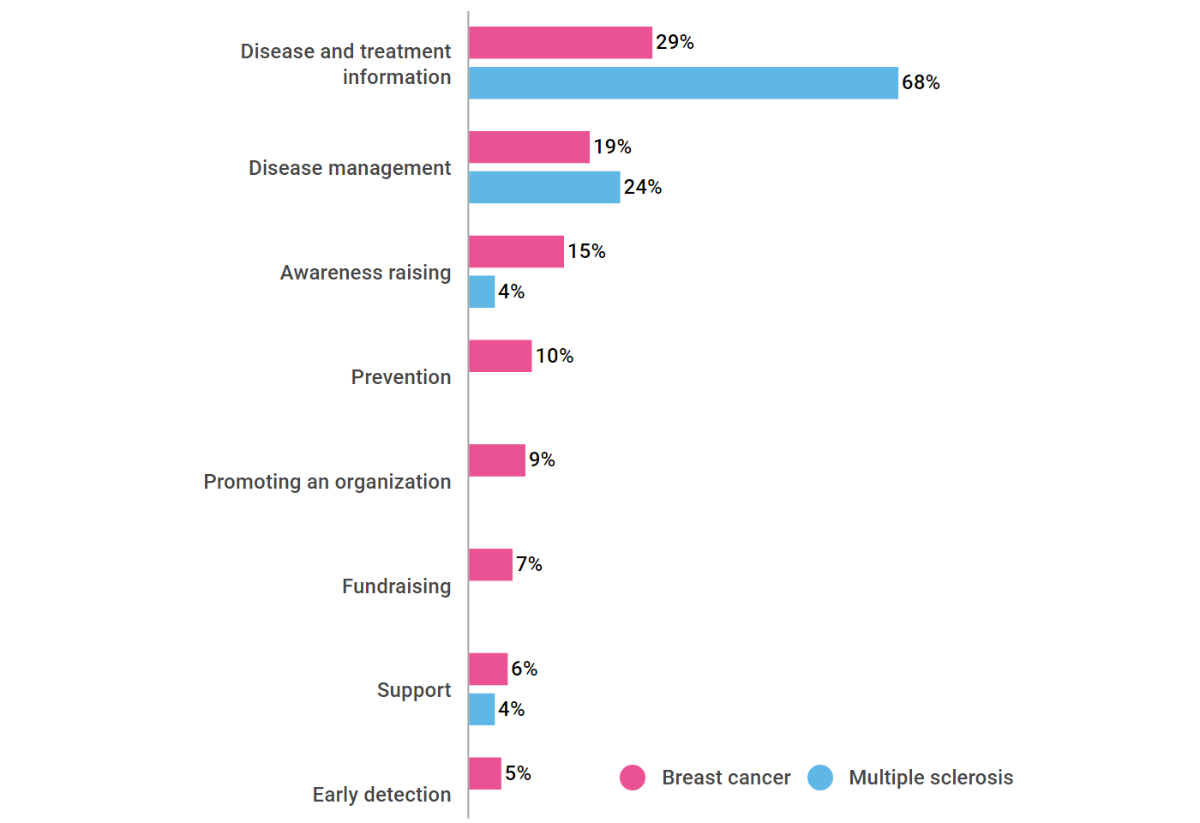
mHealth app features found in embedded unit 1 for breast cancer (n=599) and multiple sclerosis (n=25).

Chronic conditions have symptoms that affect patients physically, emotionally, and even cognitively. Figure 3 shows some of the findings of the embedded unit 1 in which disease management (symptom management) and disease information were greatly represented among the available mHealth solutions.

There are lifestyle changes that persons with chronic conditions adopt that can cause sometimes even more resistance and problems than simple medication adherence. In embedded unit 3, MS conditioned the way persons with MS lived their life, seeing their physical energy as a resource that needs to be managed and in many other subtler ways. Simple weather conditions such as warmer temperatures worsened MS symptoms according to some of the interviewees. In some cases, chronic conditions can diminish their sense of self-efficacy; PWMS02 claimed that there are times when “you don’t know how much confidence to have in yourself.”

Having a social circle of family and friends who provide support was a determining factor for motivating persons with chronic conditions in embedded unit 3. Friends and family reminded them that “We’re not alone with our MS. There are people thinking about what they can do to help us” (PWMS08).

The way others who were not part of the close social circle behaved and reacted also determined their actions. Designers would benefit from understanding how patients deal with these changes and how it affects them as human beings.

The illustrative design questions for this section and their audit trail summary are presented in Textbox 2.

#### Patient Self-Narrative

The way the particular condition manifests itself in the life of the patient changes greatly how they relate to it. A person living with a condition since childhood is more likely to see it as part of themselves as opposed to thinking it is something that happened to them. Additionally, the way in which the condition manifested also plays a role, as receiving the diagnosis due to an emergency situation or routine testing changes perspectives and expectations.

In embedded unit 3, health care professionals and persons with MS commented on the different strategies that the chronic condition forced patients to undergo to appear “normal.” For example, one patient referred to strategies to cover up symptoms from others:

I use one trick, I move all my appointments to the morning so people around me don’t realize that I’m not well. I then take a break in the afternoon and if someone wants to do something, I just say that my calendar will free up again in the evening.

PWMS02

**Model component overview of the 3MD for Chronic Conditions.**

1. **Condition specific**
  Factors that act as the foundation of the design process because they provide direct and indirect knowledge about intended users, relevant stakeholders, and their characteristics.
  - Common condition problems
  - Patient self-narrative
  - Care process
2. **Motivation related**
  Factors that nourish our understanding in regard to the type of intervention and experience we are building.
  - Behavioral change aspects
  - Gameful aspects
3. **Technology based**
  The different technological factors that can be used to mold and craft the particular mHealth solution.
  - Quantification
  - Tailoring
  - Representation

**Common condition problems illustrative questions.**

**Symptom and treatment related (embedded units 1 and 3)**

- What does the medical literature say are the main symptoms of the condition?
- What kind of treatment are people with the condition receiving?
- What are common side effects for these treatments?
- What do people with the condition feel about their treatments?
- How much do people with the condition feel they understand their condition?

**Condition-driven lifestyle changes (embedded units 1 and 3)**

- How much has the lives of people with the condition changed because of the condition?
- What kind of things does a person with the condition “have to do” now?
- How has routine been disrupted for those with the condition?
- What strategies have people with the condition developed to cope?
- How can our design make people with the condition feel more in control?

**Social impact (embedded units 1 and 3)**

- How has the condition changed the way people with the condition relate to others?
- Are there things that someone with the condition feels they have to hide?
- How are the individual and social circle adapting to the changes brought on by the condition?
- In what way are people with the condition involving others in condition-related issues?
- Has living with the condition affected the relationship with significant others?

The concept of “normalcy” was very important to persons with chronic conditions. Some conditions require almost constant care; the level of disruption to normal life determines the burden of the condition. The health care professionals in embedded unit 3 often recommended understanding the emotional and psychological mindsets that the special circumstances of living with a particular chronic condition places on patients.

Another aspect to consider is what views the larger society and culture hold toward the condition in particular. A special kind of health app was present in embedded unit 1: those for raising condition awareness. Some diseases have different status within the collective mind. People with cancer are a good example: as they get better, they become survivors and command a certain level of respect because they have “beaten” the condition. However, not all cancers are treated the same way. Although breast cancer—perhaps due to awareness campaigns or the target population—is perceived as something that “happens to” women, lung cancer—due to its association with tobacco and smoking—is seen as something that the individuals “brought on themselves.”

The illustrative design questions for this section and their audit trail summary are presented in Textbox 3.

#### Care Process

The findings from embedded unit 3 were also in line with the idea that designers of mHealth solutions should acknowledge the place the intervention will have within the accepted care flow. Health care is a team effort, each stakeholder is a member with a special role to play. Chronic conditions present scenarios that require joint collaboration from many disciplines and agents, which increases the complexity and the number of stakeholders involved. The absence of health care professionals’ involvement in health app development was evident in embedded unit 1 as well as recognized in the literature.

Each and every stakeholder carries their own agenda, their own goals, and expectations and it is important to keep this in mind or the health care team can become a barrier for mHealth solutions as seen in embedded unit 3:

It’s maybe true that we [health care professionals] are not likely to recommend or suggest technology-based solutions. I never thought about it. Maybe because there is still no clear answer as to how apps can help. Perhaps we feel that the personal relationship that we form with our patients is not something we can replace with technology.

HP05

The illustrative design questions for this section and their audit trail summary are presented in Textbox 4.

### Motivation Related

Motivation derives from the French word “motivé,” which points to the concept of needs, desires, wants, or drives that we as humans may have. The design of a behavioral change mHealth intervention aims to create a solution that can motivate people to enact our intended action.

The motivation-related component describes factors that address how to influence behaviors in an engaging manner. As a result of thematic analysis, these factors were clustered as behavioral change aspects and gameful aspects.

**Patient self-narrative illustrative questions.**

**Sociocultural perspectives (embedded units 1 and 3)**

- How is the condition perceived by society?
- Do people with the condition carry any social stigma?
- How is society working to help people with this condition?
- How much condition awareness exists in society?
- Are there special accommodations required for people with the condition?

**Living with the condition (embedded units 3 and 4)**

- At what age is the condition usually diagnosed?
- How does the condition manifest for the first time?
- Do the condition and treatment regimens change over time?
- Are there different phases or stages to the condition?
- How long has the target population been living with the condition?

**Condition burden (embedded units 3 and 4)**

- How much time per day does someone with the condition have to invest in symptom management?
- Do people with the condition have other chronic conditions to manage as well?
- How much skill does disease management require?
- Are there things that someone with the condition could do before and now they cannot do anymore? How do they feel about these changes?
- In what ways do people with the condition feel that the condition disrupts their normal life?

**Care process illustrative questions.**

**Health care team composition (embedded unit 3)**

- What kind of health care professionals are involved in the treatment of this condition?
- Who is the health care professional responsible for the overall treatment?
- How are other professionals brought into the process?
- Are there nonmedical professionals involved?
- What is the involvement of informal caregivers?

**Stakeholder dynamics (embedded units 1 and 3)**

- What is the role of each stakeholder in the process?
- How do health care professionals relate with one another?
- Do health care professionals feel that there is an overlap in activities?
- What do informal caregivers think of the care process?
- How is the care process established?

**Health care system use (embedded unit 3)**

- How often do people with this condition need to interact with the health care system? How long are these interactions?
- What are the different steps required for each interaction with the health care system?
- Are visits to the emergency room an expected occurrence for people with the condition?
- Do medical interventions require rehabilitation periods?
- Are surgical interventions required?

#### Behavioral Change Aspects

Depending on the objective of our intervention, it is likely that our behavioral change approach will be different. The scale of the intervention is as important as whether we are creating a new behavior or reinforcing an existing one. Not all interventions carry the same expectations in regards to the duration of their effects. A person with MS may need continuous reinforcing of his determination to do rehabilitation exercises, whereas a breast cancer survivor who needed to deal with chemotherapy side effects does not. Some behavioral change interventions may have specific goals for specific moments in time or do not expect that the behavior remains after a certain period.

**Figure 4:**
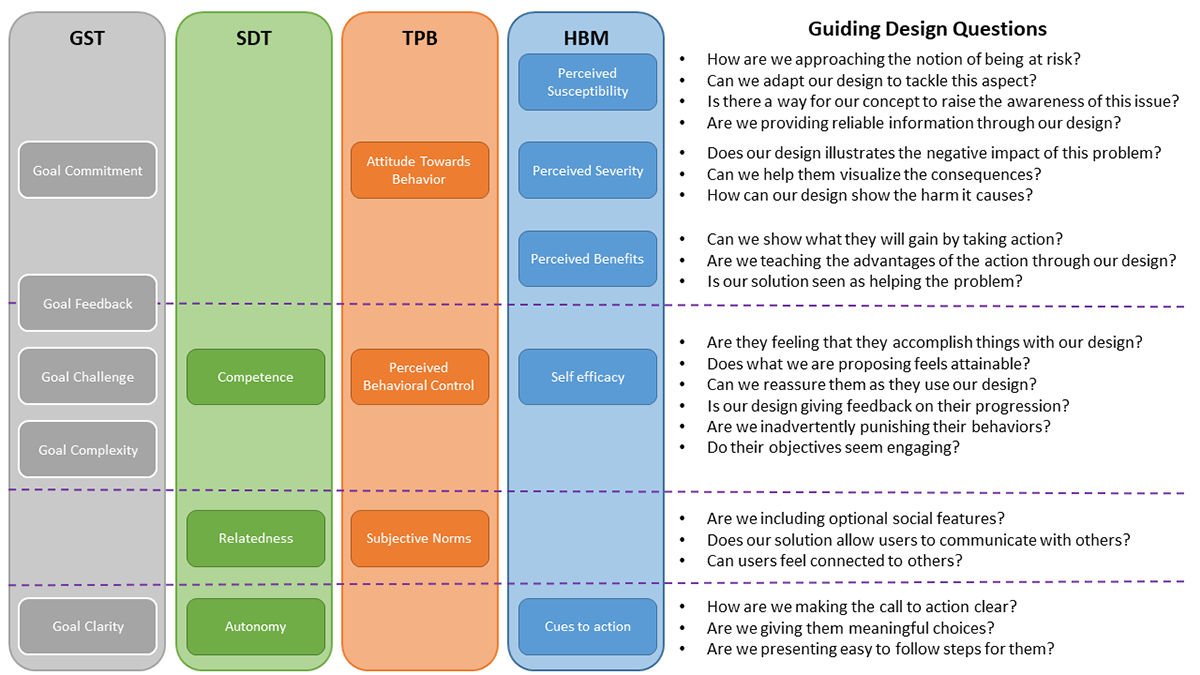
Behavioral change model requirement negotiation diagram from embedded unit 4. GST: goal-setting theory; HBM: Health Belief Model; SDT: self-determination theory; TPB: theory of planned behavior.

The related research on behavioral change states that single theory approaches are not recommended. A certain level of requirement negotiations between existing BCMs and our mHealth solution is necessary. In the embedded unit 4, BCMs were key during design negotiations. Each design concept was deconstructed to find matches with current behavioral change models; when a specific part of a BCM was not addressed by a design concept, the concept was explored further until integration with the behavioral change models felt natural or the concept was discarded. To facilitate this process, an ad hoc diagram representing the constructs from the BCMs we considered was created in embedded unit 4 (see Figure 4).

Behavioral change factors demand careful thought during the design phases to understand which behavioral change model or models to select and combine. The illustrative design questions for this section and their audit trail summary are presented in Textbox 5.

#### Gameful Aspects

The need for a more enjoyable experience was present in embedded unit 3. Game-like features were desired by persons with MS such as PWMS02 who expressed:

[an app could present something like] an obstacle course that you have to get through. [Something] that you tackle daily. The app would have to give you an alert that says you have to walk 2 km today, for example. And you have to be able to set [your own] goals. The patient should try how long he or she can walk and then perhaps increase the amount. That would maybe make people use it more. In a game, there are also tasks that you have to do. If you finish them, you get something.

PWMS02

This game-like attitude heavily resonated in several other persons with MS and even some health care professionals:

For me, it’s important that (the app) is playful. We all remain children deep down. It should have colors, some music, and be attractive.

HP03

The findings in embedded unit 1 and embedded unit 2 tell us that the trend of gamification in health apps is strong. From the related research on gamification, we understand that the creation of a gamified system is synonymous to crafting an experience that attempts to transport users to a different, more playful mindset.

**Behavioral change aspects illustrative questions.**

**Type of behavior (embedded units 1 and 4)**

- What kind of behavior change are we as designers trying to achieve?
- What models have been successfully used for this condition before?
- Is the behavior an existing behavior or a new one?
- Does the condition go through stages?
- Is our intended intervention ethical?

**Behavior over time (embedded unit 4)**

- How complex is the behavior that our intervention is trying to establish?
- Can the intended behavior be broken down into smaller or shorter behavioral components?
- What is the estimated duration of the intended behavioral change?
- What evidence is available for the intended behavior change?

**Intervention scale (embedded unit 4)**

- What is the size of our intended population?
- Are there models that fit the size of our intervention better?
- Are there proven ways to reach our target audience?
- How are we measuring the effectiveness of our intervention?
- Which are the most cost-effective ways for the size of our intervention?

**Gameful aspects illustrative questions.**

**Experience style (embedded units 1, 3, and 4)**

- What opportunities are we as designers offering for socialization?
- Does our design provide challenging opportunities for our intended users?
- Does our experience or intervention benefit from having a narrative?
- How are we as designers providing our intended users with clear objectives?
- How rich and complex is the game world of our design?

**Immersion density (embedded unit 4)**

- How deep of a gameful experience does our intervention requires?
- Are there metaphors that can help tell a story within our design?
- How does our gameful system fit our intended target population?
- Is the tone of our message coherent with our design?

**Element selection (embedded units 2 and 4)**

- What kind of game elements can we as designers use for this design?
- Which elements seem appropriate for the type of experience that we as designers are building?
- Are virtual self-representations needed in our design?
- How can we as designers transmit the feeling of progress?
- Is social comparison useful to our design?

From the work done in embedded unit 4, it became clear that the use different types and layers of gamefulness needed to be adjusted for our particular intervention. The task of constructing a gameful experience requires that we acknowledge that not all systems require or even benefit from the same features or elements. In embedded unit 3, participants commented on the advantages of social interaction among peers, but the idea of competing with each other was not appreciated:

It’s important to distinguish how you’re connected. I don’t want to compete [with other persons with MS].

PWMS07

A more immersive system might make a symptom management app take the form of monsters invading us unless we warn them off through “rituals of prevention” that cast them off, whereas a less immersive system might only require that we check the tasks as done.

The illustrative design questions for this section and their audit trail summary are presented in Textbox 6.

### Technology Based

Mobile technologies offer a wide variety of features that can be used to help chronic patients improve their quality of life and manage their condition. Technology should work together with the condition-specific needs and engagement aspects to find a solution that fully benefits all stakeholders. In embedded unit 4, we centered the design of a mHealth solution on the intended users, following personas that we created in embedded unit 3 as user representations.

The design features for mHealth solutions for persons with MS found in embedded unit 3 are:

1. Customizable goal setting: challenges need to be tailored to the specific person with MS characteristics.
2. Energy profiles and fatigue management: information and tools that help users in managing their day-to-day activities.
3. Patient education: offer verified information that is helpful and reliable.
4. Data visualization: information must be presented in a way that is meaningful to persons with MS.
5. Positive feedback system: rewards and incentives for completing tasks and objectives.
6. Activity tracking: register metrics such as distance walked or run, calorie consumption, heart rate, and quality of sleep among others.
7. Exercise library: a collection of different activities beneficial to persons with MS such as fitness or relaxation techniques that can be selected.
8. Game-like attitude: playfulness is a mindset whereby people approach activities as something not serious, in a way that is highly pleasurable and motivating.
9. Strong evidence base: features and information offered should have a solid scientific foundation.
10. Remote monitoring: health care providers can follow persons with MS progress and give feedback.
11. Optional sociability: ability to opt-out of social media features such as messaging, feeds, and/or other kinds of social comparisons.
12. Reminders system: notifications that remind persons with MS to engage in activities.
13. Personal data management: access to personal information and data defined by the user case by case.

The technology-based component describes factors that are directly connected to the technical capabilities that mobile technologies offer that could be used in behavioral change mHealth interventions. Using thematic analysis, these factors were subgrouped as quantification, tailoring, and representation.

#### Quantification

By giving a clear numerical value to an event or activity, we are providing an objective reference point that is useful for us to make decisions. Related research on chronic conditions speaks highly of different variable tracking during the course of a disease. This was in line with the findings of embedded unit 1, where a large percentage of health apps dealt with disease management.

Gathering data can help empower people with chronic conditions and allow them to take more control over their lives; the need for this design feature was mentioned previously. The mHealth tool called More Stamina we designed in embedded unit 4 had the purpose of tracking and monitoring fatigue in persons with MS. mHealth solutions can propose systems that provide information for decision making and feedback on how they are doing and what to do. With the right tools and the right data as input, figuring out the next move for our intended users is more accessible.

The illustrative design questions for this section and their audit trail summary are presented in Textbox 7.

#### Tailoring

The majority of mobile phones are sold with embedded accelerometers, gyroscopes, and GPS chips. It is because of these features and how we use them that where we have been and things such as how often we really go to the gym can be known. There is probably more data about our lives in our mobile phones than there is in our houses. However, not everyone relates to their mobile devices in the same way. The degree to which people obtain, communicate, process, understand, and deal with electronic resources, such as the internet and other technologies—or technological literacy—plays a big role in consumer health informatics. One person with MS from embedded unit 3 phrased this as: “those who are not interested in technology would never use an app.”

It is important to keep in mind that the information gathered by mobile devices can be taken advantage in a way that improves the overall experience. If we crosscheck the fact that a cancer patient is in a specific GPS position every Thursday at 2 pm for 4 hours with the fact that this location is the oncology department of the local hospital, we could infer that they are undergoing chemotherapy. Knowing this could prove useful for recommending suitable actions for this context and patient, perhaps offering educational reading material during this period or even withholding suggestions for physical activity immediately after. Having a personalized experience reportedly helps persons with chronic conditions feel that a given solution is right for them; this was felt in embedded unit 3. In embedded unit 4, mHealth capabilities were used to learn about the user’s habits and, once sufficient information was gathered, a personalized recommendation system took over. The illustrative design questions for this section and their audit trail summary are presented in Textbox 8.

**Quantification illustrative questions.**

**Tracking (embedded units 1, 3, and 4)**

- Does the condition have parameters that need to be tracked?
- What kind of parameters are we as mHealth designers interested in following?
- Do the technological capabilities allow for direct tracking?
- How accurate are the devices we will use for keeping track?
- How reliable are the device tracking capabilities?

**Monitoring (embedded units 3 and 4)**

- Does remote monitoring help the care process?
- How can we as mHealth designers enable health care professionals to follow the target population?
- Are intended users able to control what is being monitored?
- Are communication channels offered to health care professionals to contact the intended users?
- What role are we offering for the social circle within our design?

**Feedback (embedded units 1, 3, and 4)**

- How are we as designers letting intended users know of their progress?
- In what ways does our designed system notify intended users about corrective actions?
- How are we as mHealth designers encouraging intended users to perform the desired actions?
- What metrics are we as mHealth designers providing the intended users?

**Tailoring illustrative questions.**

**Context awareness (embedded units 1 and 4)**

- How are we as designers using the technological capabilities to learn about our intended users?
- What kind of information can we as designers learn directly through the device? Are there ways to indirectly gain more information?
- Does the use of geolocalization provide us as mHealth designers with useful insights?
- Would using social media information benefit our understanding of the situation of the intended users?

**Just-in-time recommendations (embedded units 1 and 4)**

- How can we as designers find the right moment for making a recommendation through technology?
- Are there different levels or types of actions that we as designers want the intended users to take?
- What kind of prompting do we as designers want to provide our intended users?
- How do we as designers evaluate the validity of our suggestions to intended users?

**eHealth relationship (embedded unit 3)**

- What do intended users feel about technology in general?
- Do intended users seek out online health information?
- How likely are our intended users to use technology to help them with their condition?
- Do intended users feel they have enough skills to use the technology?
- What causes our intended users to start and stop using a technological solution?

#### Representation

As methods for tracking all sorts of patient-related data are continuously being developed, we need to find ways in which this information is presented in a meaningful and relevant way to patients. Data by itself does not provide valuable insights on its own: it must be gathered, organized, made interpretable, and then analyzed to be of any use. Turning statistics into actionable information is what makes a difference.

In embedded unit 3, the health care professionals who worked with persons with MS said that it was often helpful to have some sort of visual representation to aid in the education and rehabilitation. The patients themselves also viewed this as important; for instance, PWMS06 felt that:

[In general, if you want] to convince people that physical activity is the key, we need to give them targets. Having feedback to how you are doing is good. We need to know we are doing something right.

PWMS06

Representing information in didactic ways allows persons with chronic conditions to see connections with their actions and better interpret data. The mHealth solution designed in embedded unit 4 represented the patient’s overall energy through a progress bar composed of Stamina Credits, a unit we devised to quantify the estimated effort an activity might take. In this manner, persons with MS had a more tangible notion bridging the gap between the abstract concept of “energy” to a representation of the actual experience at the end of the day. The illustrative design questions for this section and their audit trail summary are presented in Textbox 9.

### Model Summary

The work from embedded unit 1 provided a clearer picture of the current landscape for mHealth solutions for chronic conditions. Through these studies, the intended purposes, content reliability, and involved stakeholders in the development of these health apps is now known. These studies also hinted at the current gamification trend in mHealth for motivating users, which was explored in embedded units 2 and 3.

The 3MD for Chronic Conditions follows user-centered philosophy, in line with the work of embedded units 3 and 4, to take into account the perspectives from the different stakeholders involved in the care of a chronic condition, as well as the dynamics and elements that can create behavioral change. In embedded unit 4, the requirement negotiation between the medical knowledge, BCMs, and gamification was integrated and fostered the construction of this model.

**Figure 5:**
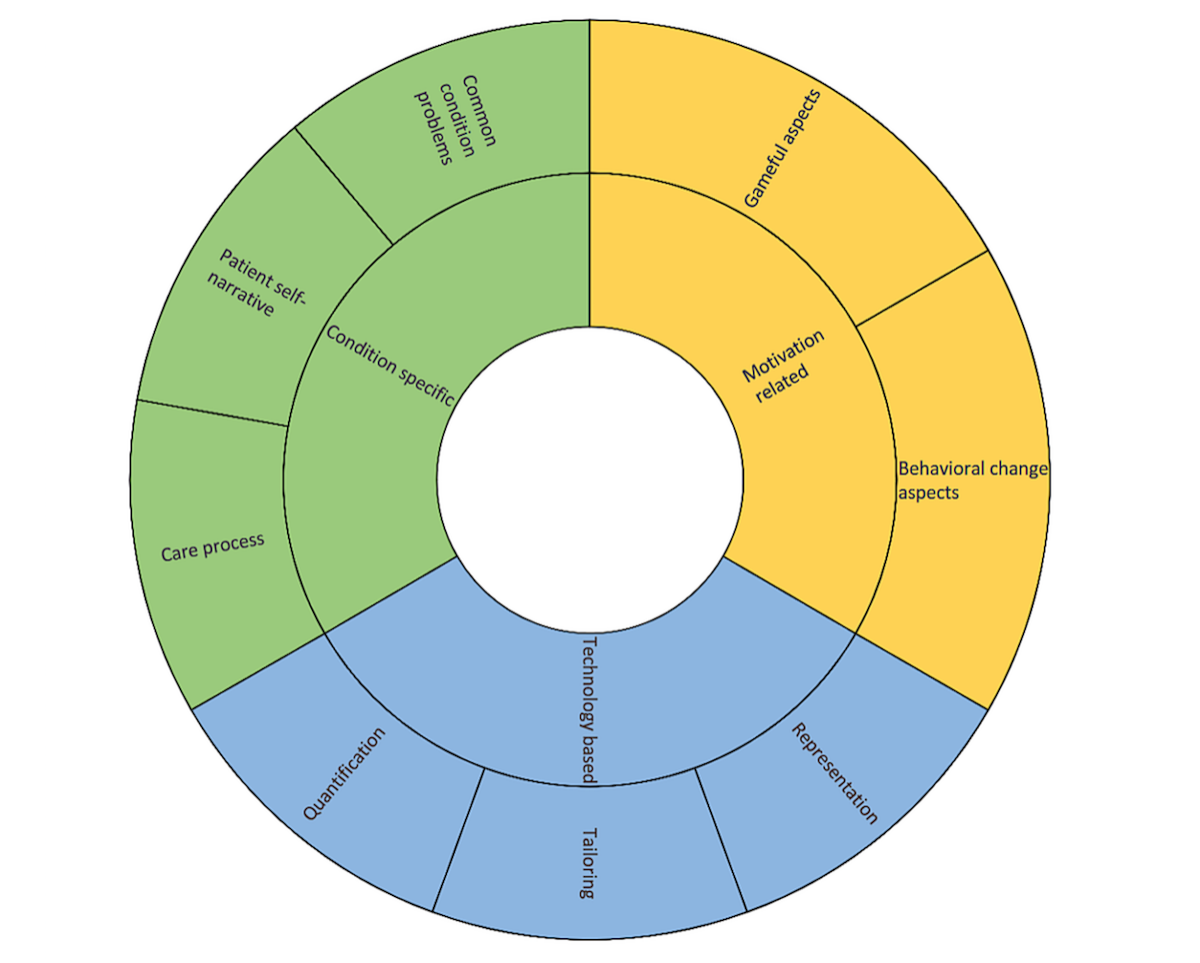
Factors of the Model for Motivational Mobile-health Design (3MD).

Figure 5 shows the design factors of the 3MD. Designers are suggested to use the different groups of illustrative questions for inspiration and to make sure that no key element is left behind in their design.

The components of the 3MD for Chronic Conditions nurture and build on top of each other and are interconnected and interdependent. A condition-specific issue can affect our choice of behavioral change model in the same way that the selection of a technology-based issue can alter the overall experience. A negotiation between all components must happen so that these factors properly align to produce our mHealth solution. Further, the model has been intentionally developed in a way that allows it to be used simultaneously with other existing frameworks for design and analysis.

**Representation illustrative questions.**

**Didactic (embedded units 3 and 4)**

- Are we as designers expressing the information in a friendly manner?
- How are we making the call to action clear to our intended users?
- Are we as designers presenting easy to follow steps for the intended users?
- Can we as designers break down the information in smaller and easier to comprehend segments?

**Dynamic (embedded units 3 and 4)**

- How are we as designers taking advantage of the mHealth technological capabilities to communicate our meaning to our intended users?
- How are we accounting for different learning styles in our intended users?
- What kind of metaphors or analogies can we as designers use facilitate comprehension?
- Can we as designers use animations or simulations to represent key concepts?

**Meaningful (embedded units 3 and 4)**

- Are we as designers giving the intended users meaningful choices within our design?
- Can we as designers show how each action is personally connected to our intended users?
- Does our design highlight the benefits for the intended users?
- Are we as designers setting realistic expectations for the intended users?
- How can we as designers make the experience more relevant to our intended users?

Designers are to view this model as a tool to help guide how to approach the problem of designing behavioral change mHealth solutions for chronic conditions. During the design process, designers are encouraged to use the illustrative questions as items to explore and find answers within their own project. These questions prompt the designers to understand what may be missing in their design and needs to be addressed.

## Discussion

### Principal Findings

The main contribution of this work lies in that it proposes a model that is unique because it demonstrates how different disciplines can be combined in a meaningful way to address a gap in the current body of knowledge. It also furthers the understanding of what to consider and to explore in the design of new behavioral change mHealth solutions.

The proposed model addresses the gap in the current body of knowledge regarding the combination of condition-specific knowledge with the understanding of technological opportunities and human factors. This research combines these three factors, which have been studied as separate elements in previous research, and shows how this integration reveals new and meaningful aspects about designing mHealth solutions for chronic conditions. Behavior change support systems are a relatively new area of research; therefore, theoretical efforts made for promoting scientific research in the area are valuable.

The proposed model can help designers to understand factors for the design of mHealth solutions and it offers illustrative design questions that can be used by mHealth designers from different disciplines to recognize and integrate factors relevant in designing mHealth solutions for chronic conditions.

### Comparison With Prior Work

Health IT for the promotion of healthier lifestyles seems to be one of the most prominent areas for the future of health care [115] with this area receiving increasing attention from the technological sector and investors [19,116]. The need for health IT solutions to be engaging has been repeatedly highlighted in the literature [117,118] because evidence-based interventions are significantly more impactful [27]. Behavioral theory and UCD have widely acknowledged merits in their application to digital health interventions. Many have underscored the need for digital health interventions to be grounded in behavioral theory [17,119,120], designed with in-depth understanding of the target population [121], and developed involving the relevant stakeholders [122,123]. The 3MD for Chronic Conditions is the first to identify how health behavioral change theories and gamification can be used for user-centric design of mHealth solutions for persons with chronic conditions. The components and themes presented in this model emerged from explorations of the mHealth state of the practice, frequently used BCMs, and interactions with persons with chronic conditions and their caregivers.

There are current trends in the design of health IT that point out how solutions should engage users in meaningful ways [117,118] and understanding what the intended users go through helps the design process. Patient experience has been recognized as one of the pillars of quality in health care, along with clinical effectiveness and patient safety [124]. This experience is their personal interpretation of the service process and how they related to it during the course of each interaction [125]. These methods are largely inspired by the field of human-centered design in which the user perspective is seen as a central component to the design process [126]. The concept of the patient journey describes all the sequential steps in providing a patient’s care, including clinical and nonclinical steps.

The 3MD for Chronic Conditions takes into consideration the context of the person with a chronic condition and its narrative. Understanding the context of a person is useful to approach their problems in a more integrative way. White and Epston [127] proposed that we create meaning by structuring our experiences into stories, which are accounts of our lived experiences put in sequence across time. The story provides a sense of continuity and meaning as it gives the past a history, it brings order to the present and attempts to predict a future. There is an emerging narrative that occurs from living with a condition. This narrative provides meaning, context, and sometimes perspective for the patient’s situation. Disease management is an ongoing situation for these conditions and transitioning into this new normal is quite a problem. The new normal state may comprise rehabilitation, oncological treatment, insulin management, physiotherapy, or sometimes even palliative care. Reaching a normalcy state is key for living with chronic conditions; patients do their best to see themselves as essentially normal persons leading normal lives [128]. Normalizing the situation becomes a necessity and several mechanisms play a role. It is common practice for people in these situations to engage in acts of covering up behaviors, including minimizing physical activity to hide fatigue symptoms, desensitization measures such as making fun of their own disability, and making trade-offs, such as accepting less desirable circumstances just to be able to do what they wanted to do [129]. The 3MD places a strong focus in understanding the context of the target population and the intended intervention. This is in line with what has been stated in the literature as fundamental in the development of behavioral change digital health interventions [48,74]. Other design models such as the PSD [74] and the IDEAS model [75] agree on the importance of fully understanding contextual factors, but are not specific for mHealth solutions or chronic conditions.

The 3MD for Chronic Conditions was intentionally designed to be agnostic to specific BCMs making it easier to adapt to different theories as needed. There are many recommendations to use multiple theories for health behavioral change [71]; by prompting reflection on the intended behavioral intervention aspect, the model favors an integrative approach.

As a model for mHealth solutions design, 3MD for Chronic Conditions places particular consideration on the capabilities and challenges that mobile technologies offer. When building mHealth solutions, there is a variety of settings and possibilities that need to be accounted for. For example, a solution that supports an existing chronic disease management program differs from a standalone app in many ways [76]. The Chronic Disease mHealth App Intervention Design Framework [76] integrates clinical and behavioral change evidence for intervention and feature design, but is not focused on the users and their needs. In the same manner, it does not address the issue of engagement.

In many behavior change interventions, technology is still used as a passive medium that mostly serves to expedite the process of communication with the user. Behavioral change interventions that take advantage of the mobile capabilities can rapidly adapt based on the individual’s current and past behavior and situational context [17]. The concept of “just-in-time” of Intille et al [130] is used to characterize interventions that adjust based on data obtained during the intervention. Additionally, standard health interventions have to consider the capacity of the intended users to process and understand basic health information, called health literacy [131]; mHealth designers need to also consider how technologically literate their users will be [105]. The 3MD for Chronic Conditions acknowledges the importance of these issues and specifically presents aspects to address them in a manner that can provide valuable insight for designers.

Behavioral change interventions have been identified as potential areas for the application of gamification [48,132]. The 3MD considers gameful design as an engagement tactic that can be tailored for the target population, the intended intervention, and the type of experience we are trying to achieve. Gamified systems have been described as complex interventions on themselves; the overreliance on points systems and disregarding contextual factors have led to unsuccessful gamification [92,133]. Thorough analysis of the content, structure, and delivery of the intervention and its components is needed for a desirable outcome [134,135]. Gamification design frameworks such as Werbach and Hunter’s [89] or the Octalysis [90] provide notions that can be helpful for designers, but were not created with health care scenarios in mind. Health-oriented gamification frameworks such as the Wheel of Sukr [92] or PACT [93] exist and, although useful, they focus on diabetes care and rehabilitation systems, respectively. The 3MD for Chronic Conditions aims to go beyond one particular condition and into chronic condition care. The components presented in this model provide a conceptual way to help approach the challenges that designing an engaging behavioral change mHealth solution for chronic conditions poses.

Unlike other available design frameworks that are used to explain or describe how mHealth design should be embarked on, the proposed model also offers a series of illustrative design questions that can be used by designers to better understand the problem at hand and how to address it. The need for more concrete guidance in mHealth design has been highlighted often [75,136] and can be particularly important, keeping in mind that the bulk of consumer health informatics mHealth solutions seem to be designed by small companies and entrepreneurs [100,101].

Finally, the absence of health care provider involvement in the design of health IT has been raised in many occasions [137-141] is addressed within this model. Active involvement and participation from all relevant stakeholders are contemplated in the design process through the use of 3MD for Chronic Conditions.

### Limitations

This work should be interpreted in the context of its limitations, which are discussed subsequently.

There are inherent limitations to the embedded case study methodology. The features that case study methodology offers that provided the rationale for its selection, also present certain limitations in its usage. Some authors [142] have commented how case study methodology may lack representativeness, rigor in data collection, construction, and analysis of the empirical materials. As the investigator, I was the primary instrument of data collection and analysis; therefore, my subjectivity is vulnerable to the problem of bias. However, the issues often raised against qualitative studies are so only in light of certain epistemological views. Qualitative approaches take into account and include differences; they do not attempt to eliminate what is inherent to being human and cannot be discounted [96,142,143]. During idea generation, designers also use their background experiences and skills, as well as different types of internal and external stimuli they might have access to [144]. Further, the audit trail is provided to help increase transparency.

The use of methodological triangulation opens the possibility of disharmony based on conflicts of theoretical frameworks and differences in the epistemologies of each method used in subcases [145]. However, the findings from these methods were considered as different parts of a knowledge continuum in line with Foss and Ellefsen [146], aiming to improve the accuracy of my findings and to increase their scope [94,95].

Additionally, this model is the outcome of mHealth design exploration in which only two conditions were considered, which leaves room to question the generalizability of its results. Another important aspect is that the model does not consider the fact that there are chronic patients who live with more than one chronic condition. Multimorbidity, as this phenomenon is called, is known to impact on health care costs and resources across health systems, regions, disease combinations, and person-specific factors (including social disadvantage and age) [147]. However, this was so because the creation of the model was driven by the different cases that I had the opportunity to work on and there was no mention of other concurrent conditions. Even so, the way the model is conceived allows for generation of further subthemes within the components that could accommodate multiple conditions.

From a design perspective, the proposed model uses experiences that stem from a single design case in which the design evaluation did not involve the intended end users. However, the model was systematically constructed based on the different studies and related work and, because it follows empirical evidence, its results are still valid.

Finally, as this model uses gamification in one of its factors, it is possible that in the future the use of game elements in health trend will turn out to be just a fad [29], and this subcomponent could lose its relevancy. Notwithstanding, the 3MD for Chronic Conditions uses gamification as a way to further explore and enhance motivational aspects; this subcomponent could be adjusted and amended in light of future findings.

### Future Research

Future research is necessary to validate the kinds of conclusions that can be drawn from the model proposed in this paper. More empirical design studies are needed to validate the 3MD for Chronic Conditions and assess its usability. This provides a good starting point for further research regarding the use of the model in different phases of the design cycle and how it can be approached by different stakeholders. The exploration of multimorbidity in the context of the proposed model may also constitute the object of future studies.

### Conclusions

The results on this paper address a recognized gap in research and practice on how medical, design, and technology factors can guide the creation of mHealth solutions to face the global challenge that chronic conditions pose. Further, this work explores the design of behavioral change mHealth solutions for chronic conditions and proposes a model that could be of use in the generation of new tools to help chronic patients.

## Abbreviations

3MD: Model for Motivational Mobile-health Design
AIC: Akaike’s information criterion
BCM: behavioral change model
BCSS: Behavioral Change Support System
eHealth: electronic health
HP: health care professional
IT: information technology
mHealth: mobile health
MS: multiple sclerosis
PSD: Persuasive Systems Design
PWMS: persons with multiple sclerosis
ROC: receiver operating characteristic
UCD: user-centered design
